# Proximity Ligation Mapping of Microcephaly Associated SMPD4 Shows Association with Components of the Nuclear Pore Membrane

**DOI:** 10.3390/cells11040674

**Published:** 2022-02-15

**Authors:** Alexandra C. A. Piët, Marco Post, Dick Dekkers, Jeroen A. A. Demmers, Maarten Fornerod

**Affiliations:** 1 Department of Cell Biology, ErasmusMC, Dr. Molewaterplein 40, 3015 GE Rotterdam, The Netherlands; a.piet@erasmusmc.nl (A.C.A.P.); m.g.post@erasmusmc.nl (M.P.); 2 Proteomics Center, ErasmusMC, Dr. Molewaterplein 40, 3015 GE Rotterdam, The Netherlands; d.dekkers@erasmusmc.nl (D.D.); j.demmers@erasmusmc.nl (J.A.A.D.)

**Keywords:** nuclear pore membrane, sphingomyelinase, NUP35, SMPD4, n-smnase-3

## Abstract

SMPD4 is a neutral sphingomyelinase implicated in a specific type of congenital microcephaly. Although not intensively studied, SMPD4 deficiency has also been found to cause cell division defects. This suggests a role for SMPD4 in cell-cycle and differentiation. In order to explore this role, we used proximity ligation to identify the partners of SMPD4 in vivo in HEK293T cells. We found that these partners localize near the endoplasmic reticulum (ER) and the nuclear membrane. Using mass spectrometry, we could identify these partners and discovered that SMPD4 is closely associated with several nucleoporins, including NUP35, a nucleoporin directly involved in pore membrane curvature and pore insertion. This suggests that SMPD4 may play a role in this process.

## 1. Introduction

The nuclear pore complex (NPC) is a crucial component of the nuclear envelope (NE) that consists of multiple proteins spanning both leaflets of the NE. It can be subdivided into several subunits and its main function is to allow selective transport between the nucleus and the cytoplasm [1,2]. Proper formation of the NPC is essential for the cell to function, and incorrect or delayed formation can lead to cell-cycle disturbances [3,4,5,6] Post-mitotic NPC assembly occurs right after open mitosis during the reformation of the NE. This is a quick process, inserting pre-assembled subunits stored in the ER during mitosis into the reforming nuclear envelope. Interphase assembly occurs during the interphase when the nucleus grows to accommodate the doubling chromosomes. This is a slower process that requires extensive deformation of both leaflets of the NE. Both processes have been intensively studied, but many aspects remain to be elucidated [7]. Until recently, SMPD4 has not been intensively studied. SMPD4 has been classified as a sphingomyelinase, an enzyme that breaks down sphingomyelin into ceramide and phosphocholine [8,9,10]. However, unlike other sphingomyelinases, SMPD4 does not localize to the Golgi system or the plasma membrane but seems to localize to the ER and the nuclear membrane instead [8,9]. This suggests that SMPD4 may have localized functions. Additionally, Atilla et al. [11] showed that SMPD4 depletion causes severe delays in cell-cycle progression. Recently, Magini et al. [12] discovered that congenital bi-allelic SMPD4 mutation causes a severe microcephaly phenotype in humans. Patient cells displayed cell-cycle delays and rough ER deformation, while a further study of SMPD4 showed that it interacted with elements of the nuclear pore. The role of SMPD4 in the NPC, however, remains unclear.

Our knowledge about the interaction partners of SMPD4 is still very incomplete. It has been found as a prey protein in previous interactome research, where it was associated with the NE and the NPC components [13] or the centrosome [14]. However, only Magini et al. [12] used SMPD4 as bait using immunoprecipitation (IP) and mass spectrometry. Interactome studies often find different and complementary results depending on methodologies, and membrane-associated proteins may be detected more easily using proximity ligation rather than IP. The aim of this study is to find new interacting partners of SMPD4 using BioID proximity ligation and gain better insight into its function and position at the nuclear pore.

## 2. Materials and Methods

### 2.1. Cloning, Transformation, Bacterial Culture, and Sequencing

cDNA encoding SMPD3 and SMPD4 were kindly donated by Dr. Hannun [15] and Dr. Mangini, respectively [12]. 3xHA-miniTurbo-NLS_pCDNA3 was a gift from Alice Ting (Addgene plasmid # 107172; http://n2t.net/addgene:107172 (accessed on 31 October 2018); RRID:Addgene_107172). Ref. [16]. All steps of the cloning process were performed according to the protocol provided by the manufacturer. To enable cloning, restriction enzyme recognition sites were introduced into the SMPD3 and SMPD4 cDNA using overhanging primers in a 2-step PCR (Appendix B Table A2). Vectors were digested using enzymatic restriction digestion, dephosphorylated with Shrimp Alkaline Phosphatase (New England Biolabs, Ipswich, MA, USA), and ligated to purified PCR products using T4 DNA ligase (NEB). Plasmids were introduced into One Shot Stbl3 chemically competent *E. coli* (Thermo Fisher Scientific Inc., Waltham, MA, USA). For miniprep, a colony was inoculated and grown in Luria–Bertani medium with 50 µg/µL of ampicillin, and plasmid DNA was isolated using a QIAprep Spin Miniprep Kit (Qiagen, Venlo, The Netherlands). For midiprep, DNA was isolated using the NucleoBond Xtra Midi kit (Bioké, Leiden, The Netherlands). DNA fragments amplified with PCR were sequenced by the Macrogen EZ-Seq service (Macrogen, Amsterdam, The Netherlands).

### 2.2. HEK293T Culture, Transfection, and Biotinylation

HEK293T cells were cultured in DMEM supplemented with 2% penicillin-streptomycin and 10% fetal calf serum at 37 °C under 5% CO_2_. Cells were passaged by washing once with phosphate-buffered saline, followed by treatment with trypsin-EDTA at room temperature. For immunofluorescence, cells were cultured on 18 mm glass coverslips in 6 well plates. For mass spectrometry, cells were cultured in 145 mm plates. Cells were transfected at 80% confluency using 12.5 µL/mL Lipofectamine2000 and 3.75 µg/mL plasmid in optiMEM, using 800 µL total volume for 6-wells and 10 mL total volume for 145 mm plates. DNA-lipid complexes were replaced with serum containing medium after 4 h. Biotinylation was induced 24 h after transfection by adding 100 mM biotin in DMSO to the culture for a final concentration of 500 µM biotin. After 30 min, cells were washed with 4 °C PBS to stop biotinylation. All cell culture reagents were obtained from Thermo Fisher.

### 2.3. Immunofluorescence

For immunofluorescence, cells were washed 3 times with ice-cold PBS before fixation with 3.7% formaldehyde in PBS for 15 min. Cells were permeabilized with 0.1% triton in PBS for 10 min and blocked with 1% BSA in PBS for 20 min. Cells were incubated with primary antibodies in the dark for 1 h, after which cells were washed 3 times with 1% BSA in PBS. Cells were then incubated with secondary antibodies for 30 min, after which cells were washed 4 times with PBS. After washing, cells were air-dried for 10 min before being placed on Thermo Scientific™ microscope slides with ProLong Gold Antifade with DAPI (Thermo Fisher). Samples were cured at room temperature for 16 h. Slides were imaged using a Leica SP5 confocal with a 40× objective with oil for confocal microscopy (Leica Biosystems, Wetzlar, Germany). Antibodies used and dilutions are given in Appendix A (Table A1).

### 2.4. Western Blotting

For Western blotting, cells were washed once with ice-cold PBS before harvesting. They were then pipetted loose with ice-cold PBS and centrifuged at 3000 rpm (rotor radius = 7.7 cm) for 3 min at 4 °C. The supernatant was removed. The volume of the pellet was estimated and double the volume of 2× sample buffer supplemented with 100 mM dithiothreitol (DTT) was added. Samples were heated at 70 °C for 10 min, then sonicated using a Diagenode Bioruptor^®^ fast and continuous for 10 min. Samples were loaded on SurePAGE™, Bis-Tris, 10 × 8, 4–12% 12-wells gel with NuPAGE MOPS buffer as a running buffer (Thermo Fisher). SeeBlue™ Plus2 Pre-Stained Protein Standard was used as a molecular weight marker (Thermo Fisher). After running, proteins were blotted in 2 h at 150 mA to a nitrocellulose membrane. Membranes were blocked with 5% BSA in TBST at 4 °C. Chemiluminescence was induced by combining 44.3 mL dH_2_O, 5 mL 1 M Tris-HCl pH 8.5, 500 µL 250 nM Luminol in DMSO, and 220 µL 90 mM p-Coumaric acid in DMSO with 45 mL dH_2_O, 5 mL 1 M Tris-HCl pH 8.5 and 31 µL 30% hydrogen peroxide, adding 1 mL of the resulting solution to the membrane and removing the solution after 1 min. After that, chemiluminescence imaging was performed on an Amersham™ Imager 600 (GE Healthcare, Chicago, IL, USA). Antibodies used and dilutions are given in Appendix A (Table A1).

### 2.5. Biotinylated Protein Pulldown

For mass spectrometry, cells were pipetted loose with ice-cold PBS (in an independent replication experiment, cells were washed 5 times with ice-cold PBS before harvesting) and centrifuged at 1500 rpm (1.0 RS) for 3 min at 4 °C. The supernatant was removed. Cells were resuspended in 1 mL ice-cold PBS and centrifuged at 3000 rpm for 3 min at 4 °C. The supernatant was removed and cells were lysed in 1 mL RIPA lysis buffer containing 1 tablet of Complete™ Protease Inhibitor Cocktail /20 mL and 1 mM PMSF. Cells were gently pipetted up and down. After lysation, lysates were sonicated using the Diagenode Bioruptor Plus with settings fast, 30 s on then 30 s off, for 20 min (Diagenode, Seraing, Belgium). Lysates were then clarified by centrifugation at 10,000 rpm. Further, 25 µL of clarified lysate was used for Western blotting. Pulldown was performed using 200 µL Pierce™ Streptavidin Magnetic Beads per sample, according to the protocol provided by the manufacturer (Thermo Fisher).

### 2.6. Mass Spectrometry

Proteins, while still bound to the beads, were subjected to reduction with dithiothreitol, alkylation with iodoacetamide, and then digested with trypsin (sequencing grade; Promega), as described previously [17]. Beads were incubated with elution buffer containing 80% acetonitrile, 0.2% trifluoroacetic acid, and 0.1% formic acid in dH_2_O for elution of bound peptides after on-bead digestion. Beads were then centrifuged in elution buffer at 1000× *g* for 10 min. Eluate was then transferred to a clean tube. An additional 0.3 mL elution buffer was added, and beads were heated at 82 °C for 5 min. Eluate was then transferred to a new tube. Elution was repeated 1 more time for a total of 4 fractions of eluate, which were pooled and dried by Speed Vac. Nanoflow liquid chromatography tandem mass spectrometry (nLC-MS/MS) was performed on an EASY-nLC coupled to an Orbitrap Fusion Tribid mass spectrometer (Thermo Fisher), operating in positive mode. Peptides were separated on a ReproSil-C18 reversed-phase column (Dr. Maisch; 15 cm × 50 μm) using a linear gradient of 0–80% acetonitrile (in 0.1% formic acid) for 90 min at a rate of 200 nL/min. The elution was directly sprayed into the electrospray ionization (ESI) source of the mass spectrometer. Spectra were acquired in continuum mode; fragmentation of the peptides was performed in data-dependent mode by HCD.

Raw mass spectrometry data were analyzed with the MaxQuant software suite [18] (version 1.6.5.0) as described previously [17] with the additional options ‘LFQ’ and ‘iBAQ’ selected. The A false discovery rate of 0.01 for proteins and peptides and a minimum peptide length of 7 amino acids were set. The Andromeda search engine was used to search the MS/MS spectra against the Uniprot database (taxonomy: Homo sapiens, release February 2019) concatenated with the reversed versions of all sequences. A maximum of two missed cleavages was allowed. The peptide tolerance was set to 10 ppm and the fragment ion tolerance was set to 0.6 Da for HCD spectra. The enzyme specificity was set to trypsin and cysteine carbamidomethylation was set as a fixed modification, while methionine oxidation and lysine biotinylation were set as variable modifications. Both the PSM and protein FDR were set to 0.01. In case the identified peptides of two proteins were the same, or the identified peptides of one protein included all peptides of another protein, these proteins were combined by MaxQuant and reported as one protein group. Before further statistical analysis, known contaminants and reverse hits were removed.

Alternatively, peak lists were automatically generated from raw data files using the Mascot Distiller software (version 2.1; MatrixScience, Boston, MA, USA). The Mascot search algorithm (version 2.2; MatrixScience) was used for searching against the Uniprot database (taxonomy: Homo sapiens, release February 2019). The peptide tolerance was set to 10 ppm and the fragment ion tolerance to 0.8 Da. A maximum number of 2 missed cleavages by trypsin were allowed. Carbamidomethylated cysteine was set as a fixed modification and oxidized methionine and biotinylation of lysine were set as variable modifications. The Mascot score cut-off value for a positive protein hit was set to 80. Individual peptide MS/MS spectra with Mascot scores below 40 were checked manually and either interpreted as valid identifications or discarded.

Raw mass spectrometry identification files containing the data of the two independent experiments are available under accession.

Magnitudes and amplitudes of proteins were calculated using the formulas M = log2(x + 1) − log2(y + 1) and A = 0.5(log2(x + 1) − log2(y + 1)) in which x and y are emPAI scores of accessions in the samples to be compared. MA plots were generated in Rstudio. Annotation scores for every accession found were retrieved from Uniprot. Accessions with annotation scores of 4 out of 5 or less were excluded.

Localisation analysis for enriched proteins was performed using Metascape [19]. Enrichment of Gene Ontology Cellular Components was assessed using a minimal overlap of 3 and at least 1.5 fold enrichment.

## 3. Results

### 3.1. Biotin-Tagged Proteins Localize at the Nuclear Membrane

**Figure 1:**
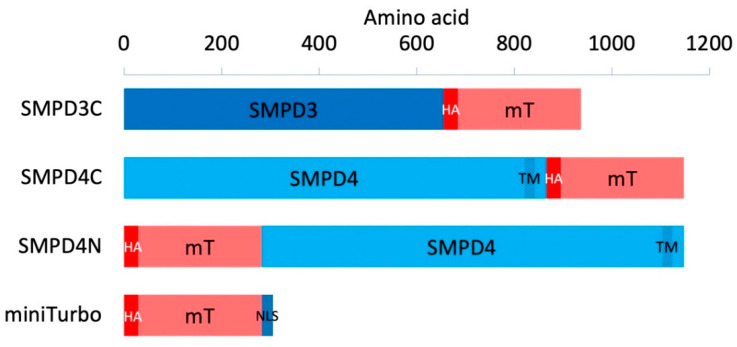
Structure of the expression constructs. SMPD3 or SMPD4 cDNAs or a nuclear localization signal (NLS) were tagged C- or N-terminally, as indicated with minisub (mT). The horizontal axis indicates amino acid position. TM: Predicted transmembrane spanning region of SMPD4 [8], HA, HA1 tag.

In order to study the localisation and interacting partners of SMPD4, we used a more efficient version of BioID called miniTurbo, created by Branon et al. [16]. This variant of BioID greatly reduces the time needed to tag proteins in its proximity with biotin, going from 24 h to less than 30 min, thus providing a more precise measurement of interaction. We created both C- and N-terminally tagged SMPD4 fusion constructs (henceforth called SMPD4C and SMPD4N), and also used the Golgi and PM localized SMPD3 [20,21], C-terminally fused (henceforth called SMPD3C), and a nuclear localisation signal (NLS) tagged miniTurbo construct (henceforth called miniTurbo) throughout our experiments as controls (Figure 1).

**Figure 2:**
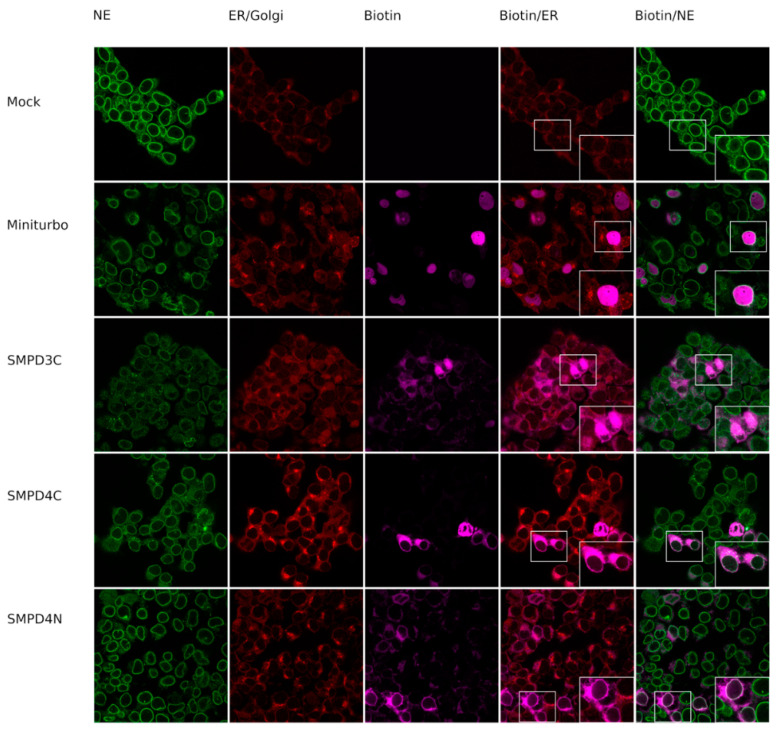
Localization of biotin in biotin ligase fusion protein-expressing (Miniturbo, SMPD43C, SMPD44C, SMPD4N) or mock-transfected (Mock) HEK293T cells. Biotin was visualized using Streptavidin-Alexa700 (magenta), nucleoporins (NE marker) with mAb414 (green), and the ER with marker BCAP (red).

In order to determine whether our constructs successfully biotin-tagged proteins in their proximity, we transfected HEK 293T cells with constructs and added biotin for 30 min, 24 h after transfection. Cells were subsequently fixed and incubated with NPC marker mAb414, ER/Golgi marker anti-BCAP31, and fluorescently tagged streptavidin to ascertain the subcellular localization of biotinylated proteins (Figure 2).

As expected, proteins biotinylated by the NLS-miniTurbo construct are mainly confined to the nucleus. The proteins biotinylated by the SMPD3C construct were found in the cytoplasm with some staining of the cell membrane, confirming earlier findings that SMPD3 localizes to the endoplasmic reticulum (ER), Golgi system, and the cell membrane.

The proteins biotinylated by the SMPD4N construct also localized to the cytoplasm with also some distinct staining of the nuclear membrane, suggesting that they reside near the ER and the nuclear membrane. The proteins biotinylated by the SMPD4C fusion protein also stained the NE, with additional staining in the cytoplasm.

### 3.2. The SMPD4 Interactome

**Figure 3:**
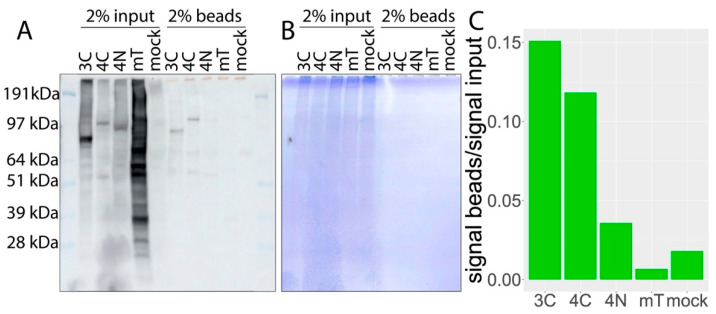
Biotinylated proteins are enriched using streptavidin-coated beads. Where 2% of cell lysates before pulldown are compared to 2% of lysates on beads after pulldown. (**A**) Membranes are stained with Streptavidin-HRP. Overexpressed SMPD4 bait protein constructs are self-biotinylated, and C-terminally tagged SMPD4 and N-terminally tagged SMPD4 constructs can be detected both before and after pulldown. (**B**) Gels after blotting. Cell lysates contain an equal amount of proteins before pulldown. After pulldown, no protein can be detected with Coomassie staining. (**C**) Streptavidin-HRP activity from lanes with beads is compared to activity from lanes with pre-pulldown cell lysates. SMPD3C (3C); SMPD4C (4C); SMPD4N (4N); miniTurbo (mT).

We next wished to identify proteins biotinylated by miniTurbo-tagged SMPD4. For this purpose, cell lysates from transfected HEK293T cells were incubated with magnetic streptavidin beads to pulldown all biotin-tagged proteins, stringently washed, and then eluted. As shown in Figure 3A, biotinylated proteins were greatly increased in miniTurbo construct transfected cell lysates and streptavidin-pulldown compared to control (mock). In order to determine whether the enrichment of biotinylated proteins was successful, SDS-PAGE gels were colored with Coomassie, showing a greatly reduced amount of protein in the eluates after washing, while a considerable amount of biotinylated protein remained (Figure 3B,C).

**Figure 4:**
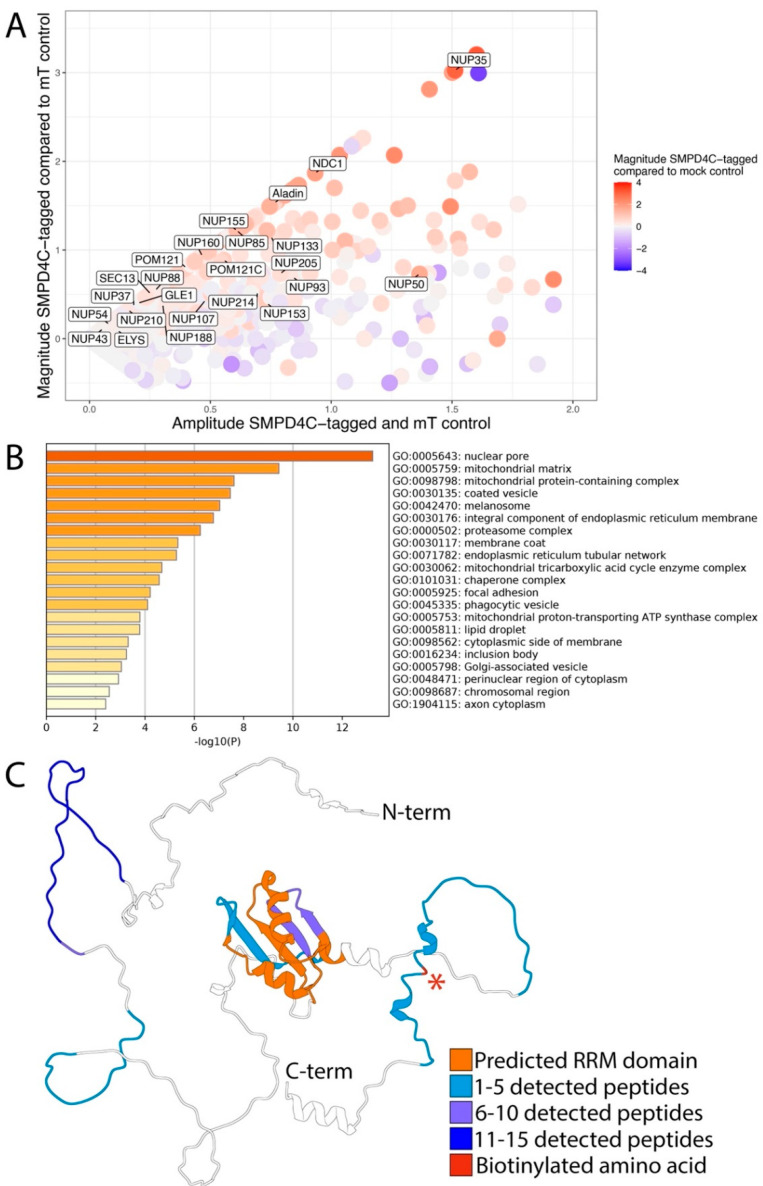
Several nuclear pore complex proteins, particularly NUP35, are enriched during pulldown after biotinylation by C-terminally tagged SMPD4. (**A**) The MA-plot shows the magnitude (log_2_ fold change) of proteins in sample 4C compared to sample mT on the *y*-axis, while the magnitude of proteins in sample 4C compared to the mock-treated sample is represented by the color. The *x*-axis represents the mean log_2_ emPAI signal (**B**) The bar plot shows the Gene Ontology analysis of proteins that were at least 1.4 (2^0.5^) fold enriched in sample 4C compared to the mock-treated sample. Data derived from one experiment, which is also representative of the biological replicate, is shown. Figure generated on https://metascape.org (accessed on 5 February 2022). (**C**) Position and the number of the NUP35 peptides (biological replicate experiment) with an asterisk indicating the lysine amino acid upon which a biotin group was detected after peptide elution with organic solvents.

In order to identify the proteins in the eluate, we used tandem mass spectrometry. After protein identification, we calculated the enrichment of proteins in our SMPD4 bait protein transfected samples compared to the control pulldowns using the exponentially modified protein abundance index (emPAI) score (Figure 4), which is linearly correlated to the abundance of protein in a sample [22].

We selected proteins that were at least twofold enriched in either the SMPD4 N-terminus or the SMPD4 C-terminus pulldowns compared to the NLS-miniTurbo pulldown. We excluded proteins with an annotation score of less than 5 out of 5 in the UniProt database [23]. Because cell lysates were not treated with RNase prior to pulldown, we also excluded RNA binding proteins, as these proteins tend to non-specifically bind to RNA binding to beads through electrostatic interaction. The resulting list (Supplementary Table S1) consisted of 112 enriched proteins for the SMPD4 C-terminus construct and 189 enriched proteins for the SMPD4 N-terminus. As shown in Figure 4, nucleoporins are markedly enriched among the proteins biotinylated by C-terminally miniTurbo-tagged SMPD4. Interestingly, the most strongly enriched nucleoporin was NUP35 (8.1 fold), followed by NDC1 (3.7 fold) ALADIN (2.9 fold), NUP155 and NUP85 (2.3 fold), and NUP133 (2.2 fold). Mostly, these nucleoporins were also detected with N-terminally tagged SMPD4, but with consistently lower enrichment scores (Supplementary Table S1). Nucleoporin enrichment, including the strong enrichment of NUP35, was reproduced in a biological replicate with more washing steps before the harvesting of cells, but an otherwise identical procedure. In contrast to SMPD4, miniTurbo-tagged SMPD3 yielded no nucleoporin hits in one experiment and a low amount in the biological replicate.

**Figure 5:**
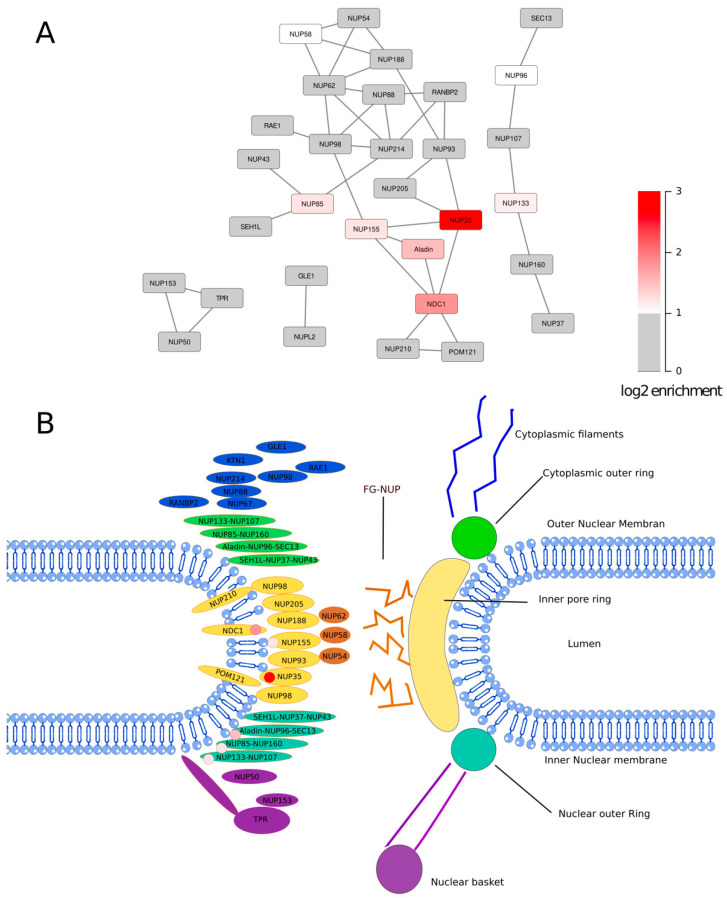
Localization of NPC proteins biotin-tagged through close proximity to SMPD4. (**A**) Network representation of nucleoporins based on published interaction studies [1,2]. Nucleoporins enriched in SMPD4-mediated biotinylation are colored in shades of red. Other nucleoporins are in gray or white (NUP58, not detected). (**B**) Schematic representation of the NPC with nucleoporins positioned at their approximate position. The red tag indicates which proteins were biotin-tagged. All tagged proteins are either components of the inner ring, the outer ring, or are transmembrane nucleoporins.

We selected proteins that were at least 1.4 (2^0.5^) fold enriched in the SMPD4 C-terminus pulldown compared to the mock pulldown for localization analysis in order to find Gene Ontology terms concerning cellular components that are enriched in proximity partners of SMPD4. Again, we excluded proteins with an annotation score of less than 5 out of 5 in the UniProt database [23] and RNA binding proteins. Nuclear pore annotated proteins were shown to be enriched in proximity partners of SMPD4, with a *p*-value of 10^−13.24^ for Gene Ontology term 0005643: nuclear pore (Figure 4B). We mapped peptides derived from NUP35 that were identified in the biological replicate to the canonical sequence of NUP35 and visualized detected peptides using the predicted protein structure of NUP35 (Figure 4C) [24,25]. Upon elution of bound peptides after on-bead digestion, we identified one biotinylated amino acid residue, outside of the predicted RNA-recognition motif of NUP35. Finally, we mapped nucleoporins associated with SMPD4 in the context of the known nucleoporin interaction network and intra-NPC localization [1,2] (Figure 5).

Interestingly, nucleoporins tagged by SMPD4 centrally cluster at the inner NPC ring component NUP35 and the pore membrane nucleoporin NCD1, while only two nucleoporins of the outer NPC ring are strongly enriched, NUP85 and NUP133.

## 4. Discussion

SMPD4 is a neutral sphingomyelinase with poorly characterized enzymatic activity, which was found mutated in a specific form of congenital microcephaly. In order to obtain more information on its cellular function, we explored the interacting partners of SMPD4. For this, we used an improved version of the BioID technique called TurboID. Magini et al. [12] have previously studied SMPD4 interacting partners using MYC-tag pulldown. There are several methods to study interactomes of proteins and they often lead to results with a partial overlap. Especially, membrane-bound proteins are difficult to pulldown and are better detected in proximity ligation experiments. As such, it is useful to study interactomes using different and potentially complementary techniques.

The BioID technique makes use of a promiscuous biotin ligase attached to the protein of interest. Once biotin is added, the biotin ligase then proceeds to biotinylate any protein in its proximity. These may be proteins that genuinely interact with the protein of interest but may also be proteins that are briefly in the vicinity of the protein of interest without truly interacting. The noise produced by these “passersby” may be mitigated by the use of TurboID and MiniTurbo who tag proteins in a 15 and 30 min window, respectively, thus reducing the number of “passerby” interactions. The short time frame, however, may lead to missing genuine interactions that happen over longer time frames. The major advantage of BioID and its derivatives is that the tagging happens in situ. Consequently, the protein will be in its normal localisation when it tags its prey with less chance of tagging proteins ex vivo. This is in contrast with complex-immunoprecipitation (Co-IP) where interacting proteins are pulled down from whole-cell lysates, thus occasionally leading to the identification of interactions that cannot possibly happen in vivo.

By using the TurboID technique, we were able to tag proteins that were in the proximity of SMPD4 with biotin. We then used this tag to either visualize these proteins in situ or pull them down and perform mass spectrometry. After visualizing the tagged proteins, we discovered that the interacting partners of SMPD4 concentrate around the endoplasmic reticulum (ER) and the nuclear pore complex. This is unlike the interacting proteins of SMPD3, which localize at the plasma membrane and the cytoplasm.

Consistent with the NE localization of SMPD4 induced biotinylation, we found several nucleoporins enriched in our mass spec analysis. Among those, the strongest was NUP35, and also nucleoporins situated in close proximity of NUP35, in particular, NDC1, Aladin, and NUP155 were biotagged. NUP35 is part of the inner pore complex and is essential for the formation of the NPC and the NE [26]. It binds directly to NDC1, a transmembrane protein, which also has a crucial role in NPC and NE formation as an anchor and is associated with Aladin [27]. NUP35 has been shown to bind directly to the nuclear membrane and to induce membrane deformation, which facilitates the formation of the NPC during the interphase [28]. NUP35 is also part of a complex with NUP205, NUP93, and NUP155, the latter of which we also found in our data. Two additional nucleoporins identified were NUP85 and NUP133, which have so far not been physically associated with NUP35.

Our findings correlate with the earlier study by Magini et al. [12]. In this study, a pulldown assay was performed using a myc-tag and then we performed mass spectrometry on the retrieved proteins. Although NUP35 was not detected as a main interacting partner of SMPD4, several other nuclear pore components were detected: TPR, SEC13, NUP133, NUP205, NUP93, and NDC1, as well as the transport receptors XPO1 and XPOT.

One biological replicate was made for mass spectrometry experiments, yielding a total of two independent experiments. Nucleoporin enrichment upon proximity ligation with an SMPD4 fusion protein has proven reproducible in the biological replicate. The SMPD4 fusion protein was overexpressed in this study, which may have caused the expression of SMPD4 in non-physiological organelles. However, previously, SMPD4 was localized at the NE [12,29] and interacted with nucleoporins [12]. This strongly suggests that our observed proximity of NUP35 and other NUPs with SMPD4 is physiological.

The cellular function of SMPD4 is not clear yet. SMPD4 is classified as a neutral sphingomyelinase, an enzyme that converts sphingomyelin to ceramide and phosphocholine. Its closest relative, SMPD3, is well studied. It is located at the plasma membrane and plays an important role in fatty acid metabolism. SMPD3 knockout has been linked to fatty acid storage defects. In contrast, SMPD4 deficiency has recently been linked to microcephaly [12] and cellular division defects [11].

Nuclear pore formation is an essential step in cell division and cell growth and defects in this process have been linked to microcephaly. There are two types of nuclear pore formation: post-mitotic and interphase formation. Nuclear pores are formed from pre-assembled subunits during post-mitotic formation. During interphase formation, however, the process is slower and proceeds in an inside-out manner that requires extensive deformation of the inner nuclear membrane (INM) and individual insertion of the nucleoporins. As mentioned previously, NUP35 has been shown to play a crucial role during the latter event by distorting the membrane, allowing individual nucleoporins to insert themselves into the NE. This raises the possibility that SMPD4 might play a role in this membrane deformation. Although SMPD4 deficiency does not change the overall levels of fatty acid in cells [11], we speculate it may do so at a local level. A local increase in ceramide could create packing effects, thus allowing NUPs to insert themselves into the membrane. Additionally, the surplus of ceramide could change the local curvature, creating the negative strain necessary for the evagination of the INM required for interphase nucleopore formation. Lastly, SMPD4 could directly recruit NUPs to the NE [30]. In order to explore these potential new roles of SMPD4, further study of the interacting partners of SMPD4 is needed, as well as an in-depth study of the exact activity of SMPD4.

## Data Availability

Raw mass spectrometry identification files containing the data used in this manuscript, including an independent replication experiment, will be available under accession.

## Supplementary Materials

The following supporting information can be downloaded at: https://www.mdpi.com/article/10.3390/cells11040674/s1, Table S1: Enriched_Proteins.

## Appendix A

**Table A1:** Antibodies used.

Antibody/Reagent	Company	Dilution
Streptavidin, Alexa Fluor™ 700 conjugate	Invitrogen	1:280
Streptavidin-HRP Conjugate	Perkin Elmer	1:5000
anti-BCAP31 (ab237485)	Abcam	1:600
mAb414 (ab24609)	Abcam	1:300
Goat anti-Mouse or anti-Rabbit IgG (H + L) Cross-Adsorbed Secondary Antibody, Texas Red or Alexa 488	ThermoFisher	1:1000

## Appendix B

**Table A2:** PCR Primers.

NotI SMPD3 forward	CTTGCGGCCGCATGGTTTTGTACACGACCCCCTTTCCT
NotI SMPD3 reverse	GTGGCGGCCGCTGCCTCCTCCTCCCCCGAAG
NotI SMPD4 forward	CTTGCGGCCGCATGACGACTTTCGGCGCCGTG
NotI SMPD4 reverse	GTGGCGGCCGCGGGCTGGTGCAGCTTCCCC
EcoRI SMPD4 forward	CTTGAATTCATGACGACTTTCGGCGCCGTG
EcoRI SMPD4 reverse	GTGGAATTCTCAGGGCTGGTGCAGCTTCCC

## References

[1] Beck M., Hurt E. (2017). The Nuclear Pore Complex: Understanding Its Function through Structural Insight. Nat. Rev. Mol. Cell Biol..

[2] Lin D.H., Hoelz A. (2019). The Structure of the Nuclear Pore Complex (An Update). Annu. Rev. Biochem..

[3] Platani M., Santarella-Mellwig R., Posch M., Walczak R., Swedlow J.R., Mattaj I.W. (2009). The Nup107-160 Nucleoporin Complex Promotes Mitotic Events via Control of the Localization State of the Chromosome Passenger Complex. Mol. Biol. Cell.

[4] Chakraborty P., Wang Y., Wei J.-H., van Deursen J., Yu H., Malureanu L., Dasso M., Forbes D.J., Levy D.E., Seemann J. (2008). Nucleoporin Levels Regulate Cell Cycle Progression and Phase-Specific Gene Expression. Dev. Cell.

[5] Fernández-Jiménez N., Pradillo M. (2020). The Role of the Nuclear Envelope in the Regulation of Chromatin Dynamics during Cell Division. J. Exp. Bot..

[6] Sakuma S., Raices M., Borlido J., Guglielmi V., Zhu E.Y.S., D’Angelo M.A. (2021). Inhibition of Nuclear Pore Complex Formation Selectively Induces Cancer Cell Death. Cancer Discov..

[7] Otsuka S., Ellenberg J. (2018). Mechanisms of Nuclear Pore Complex Assembly—Two Different Ways of Building One Molecular Machine. FEBS Lett..

[8] Corcoran C.A., He Q., Ponnusamy S., Ogretmen B., Huang Y., Sheikh M.S. (2008). Neutral Sphingomyelinase-3 Is a DNA Damage and Nongenotoxic Stress-Regulated Gene That Is Deregulated in Human Malignancies. Mol. Cancer Res..

[9] Moylan J.S., Smith J.D., Wolf Horrell E.M., McLean J.B., Deevska G.M., Bonnell M.R., Nikolova-Karakashian M.N., Reid M.B. (2014). Neutral Sphingomyelinase-3 Mediates TNF-Stimulated Oxidant Activity in Skeletal Muscle. Redox Biol..

[10] Krut O., Wiegmann K., Kashkar H., Yazdanpanah B., Krönke M. (2006). Novel Tumor Necrosis Factor-Responsive Mammalian Neutral Sphingomyelinase-3 Is a C-Tail-Anchored Protein. J. Biol. Chem..

[11] Atilla-Gokcumen G.E., Muro E., Relat-Goberna J., Sasse S., Bedigian A., Coughlin M.L., Garcia-Manyes S., Eggert U.S. (2014). Dividing Cells Regulate Their Lipid Composition and Localization. Cell.

[12] Magini P., Smits D.J., Vandervore L., Schot R., Columbaro M., Kasteleijn E., van der Ent M., Palombo F., Lequin M.H., Dremmen M. (2019). Loss of SMPD4 Causes a Developmental Disorder Characterized by Microcephaly and Congenital Arthrogryposis. Am. J. Hum. Genet..

[13] Hein M.Y., Hubner N.C., Poser I., Cox J., Nagaraj N., Toyoda Y., Gak I.A., Weisswange I., Mansfeld J., Buchholz F. (2015). A Human Interactome in Three Quantitative Dimensions Organized by Stoichiometries and Abundances. Cell.

[14] Gupta G.D., Coyaud É., Gonçalves J., Mojarad B.A., Liu Y., Wu Q., Gheiratmand L., Comartin D., Tkach J.M., Cheung S.W.T. (2015). A Dynamic Protein Interaction Landscape of the Human Centrosome-Cilium Interface. Cell.

[15] Airola M.V., Shanbhogue P., Shamseddine A.A., Guja K.E., Senkal C.E., Maini R., Bartke N., Wu B.X., Obeid L.M., Garcia-Diaz M. (2017). Structure of Human NSMase2 Reveals an Interdomain Allosteric Activation Mechanism for Ceramide Generation. Proc. Natl. Acad. Sci. USA.

[16] Branon T.C., Bosch J.A., Sanchez A.D., Udeshi N.D., Svinkina T., Carr S.A., Feldman J.L., Perrimon N., Ting A.Y. (2018). Efficient Proximity Labeling in Living Cells and Organisms with TurboID. Nat. Biotechnol..

[17] Schwertman P., Lagarou A., Dekkers D.H.W., Raams A., van der Hoek A.C., Laffeber C., Hoeijmakers J.H.J., Demmers J.A.A., Fousteri M., Vermeulen W. (2012). UV-Sensitive Syndrome Protein UVSSA Recruits USP7 to Regulate Transcription-Coupled Repair. Nat. Genet..

[18] Cox J., Matic I., Hilger M., Nagaraj N., Selbach M., Olsen J.V., Mann M. (2009). A Practical Guide to the MaxQuant Computational Platform for SILAC-Based Quantitative Proteomics. Nat. Protoc..

[19] Zhou Y., Zhou B., Pache L., Chang M., Khodabakhshi A.H., Tanaseichuk O., Benner C., Chanda S.K. (2019). Metascape Provides a Biologist-Oriented Resource for the Analysis of Systems-Level Datasets. Nat. Commun..

[20] Marchesini N., Osta W., Bielawski J., Luberto C., Obeid L.M., Hannun Y.A. (2004). Role for Mammalian Neutral Sphingomyelinase 2 in Confluence-Induced Growth Arrest of MCF7 Cells. J. Biol. Chem..

[21] Hofmann K., Tomiuk S., Wolff G., Stoffel W. (2000). Cloning and Characterization of the Mammalian Brain-Specific, Mg^2+^-Dependent Neutral Sphingomyelinase. Proc. Natl. Acad. Sci. USA.

[22] Ishihama Y., Oda Y., Tabata T., Sato T., Nagasu T., Rappsilber J., Mann M. (2005). Exponentially Modified Protein Abundance Index (EmPAI) for Estimation of Absolute Protein Amount in Proteomics by the Number of Sequenced Peptides per Protein. Mol. Cell. Proteom..

[23] The UniProt Consortium (2021). UniProt: The Universal Protein Knowledgebase in 2021. Nucleic Acids Res..

[24] Jumper J., Evans R., Pritzel A., Green T., Figurnov M., Ronneberger O., Tunyasuvunakool K., Bates R., Žídek A., Potapenko A. (2021). Highly Accurate Protein Structure Prediction with AlphaFold. Nature.

[25] Varadi M., Anyango S., Deshpande M., Nair S., Natassia C., Yordanova G., Yuan D., Stroe O., Wood G., Laydon A. (2022). AlphaFold Protein Structure Database: Massively Expanding the Structural Coverage of Protein-Sequence Space with High-Accuracy Models. Nucleic Acids Res..

[26] Hawryluk-Gara L.A., Platani M., Santarella R., Wozniak R.W., Mattaj I.W. (2008). Nup53 Is Required for Nuclear Envelope and Nuclear Pore Complex Assembly. Mol. Biol. Cell..

[27] Huttlin E.L., Bruckner R.J., Navarrete-Perea J., Cannon J.R., Baltier K., Gebreab F., Gygi M.P., Thornock A., Zarraga G., Tam S. (2021). Dual Proteome-Scale Networks Reveal Cell-Specific Remodeling of the Human Interactome. Cell.

[28] Vollmer B., Schooley A., Sachdev R., Eisenhardt N., Schneider A.M., Sieverding C., Madlung J., Gerken U., Macek B., Antonin W. (2012). Dimerization and Direct Membrane Interaction of Nup53 Contribute to Nuclear Pore Complex Assembly. EMBO J..

[29] Cheng L.-C., Baboo S., Lindsay C., Brusman L., Martinez-Bartolomé S., Tapia O., Zhang X., Yates J.R., Gerace L. (2019). Identification of New Transmembrane Proteins Concentrated at the Nuclear Envelope Using Organellar Proteomics of Mesenchymal Cells. Nucleus.

[30] Peeters B.W.A., Piët A.C.A., Fornerod M. (2022). Generating Membrane Curvature at the Nuclear Pore: A Lipid Point of View. Cells.

